# Amine Metabolism Is Influenced by Dietary Protein Source

**DOI:** 10.3389/fnut.2017.00041

**Published:** 2017-09-01

**Authors:** Soumya K. Kar, Alfons J. M. Jansman, Dirkjan Schokker, Leo Kruijt, Amy C. Harms, Jerry M. Wells, Mari A. Smits

**Affiliations:** ^1^Host-Microbe Interactomics Group, Wageningen University & Research, Wageningen, Netherlands; ^2^Wageningen University & Research Animal Breeding and Genomics, Wageningen, Netherlands; ^3^Wageningen Livestock Research, Wageningen University & Research, Wageningen, Netherlands; ^4^Netherlands Metabolomics Centre, Leiden University, Leiden, Netherlands; ^5^Department of Analytical Biosciences, Leiden University, Leiden, Netherlands; ^6^Wageningen Bioveterinary Research, Wageningen University & Research, Wageningen, Netherlands

**Keywords:** 1-methylhistidine, alpha-aminobutyric acid, amine metabolites, dietary protein source, endophenotype, metabolomics, mice models, nutritional quality

## Abstract

Growth in world population will inevitably leads to increased demand for protein for humans and animals. Protein from insects and blood plasma are being considered as possible alternatives, but more research on their nutritional quality and health effects is needed. Here, we studied the effect of dietary protein source on metabolism and metabolic amine profiles in serum and urine of mice. Groups of mice were fed semi-purified diets containing 300 g/kg of soybean meal, casein, partially delactosed whey powder, spray-dried plasma protein, wheat gluten meal, and yellow mealworm. Feed and water intake as well as body weight gain were measured for 28 days. After 14 and 28 days, serum and urine samples were collected for measurement of a large panel of amine metabolites. MetaboAnalyst 3.0 was used for analysis of the raw metabolic data. Out of 68 targeted amine metabolites, we could detect 54 in urine and 41 in blood serum. Dietary protein sources were found to have profound effects on host metabolism, particularly in systemic amine profiles, considered here as an endophenotype. We recommend serum over urine to screen for the amine metabolic endophenotype based on partial least squares discriminant analysis. We concluded that metabolites like alpha-aminobutyric acid and 1-methylhistidine are sensitive indicators of too much or too little availability of specific amino acids in the different protein diets. Furthermore, we concluded that amine metabolic profiles can be useful for assessing the nutritional quality of different protein sources.

## Introduction

If growth in the human population and food production continue at their present rate, there will be more than two billion more people to feed in 2050 and calorific deficiencies are likely to be more prevalent than they are today. One of the main concerns about the world food supply is the production of proteins for humans and livestock. In livestock sector, plant proteins (e.g., from soy) will continue to dominate in the next 10 years, but inevitably alternative sources of protein will need to be found because arable land cannot be increased in proportion. The strategy of partly replacing current protein sources for animal feed with economically viable and sustainable alternatives, e.g., protein from insects or livestock blood plasma, is one possible solution. In recent years, significant efforts have been made to introduce new protein containing feed ingredients in the diet of livestock (1, 2). Unfortunately, information on the functional properties of (new) protein sources toward their consumers, livestock production, in particular is scarce.

In general practice, animal diets are formulated based on the provision of ileal or fecal digestible nutrients and derived net energy by feed ingredients. The nutrients relate to proteins/amino acids (AAs), starch and sugars, fat, fermentable non-starch-polysaccharides, minerals, and vitamins, which can be characterized as the “strict-nutritional” value of feed ingredients. However, apart from the “strict-nutritional” value, diets and their ingredients have other “non-strict-nutritional,” functional properties in relation to, e.g., feed intake (satiety), passage rate through the gastro intestinal tract (GIT), pro- and antimicrobial properties, antioxidative and oxidative effects, immune signaling, and metabolic effects (3, 4). From the context of protein ingredients, it has the capacity to deliver AAs (both essential and non-essential) that are essential precursors for the synthesis of organic nitrogen compounds, including amines, that influence processes related to protein metabolism. Glutathione, creatinine, and nitric oxide have protective affect against oxidative stress and toxicity (5, 6). Other examples of biologically active amines synthesized from AAs are dopamine and serotonin, which are neurotransmitters exerting behavioral effects (7, 8). Further, we have previously predicted that polypeptides with antimicrobial activity, inhibition of angiotensin I-converting enzyme, as well as antioxidative, antithrombotic, and anti-amnestic activities could be generated during the digestion of several protein ingredients (9). The current practice of feed formulation for farm animals largely ignores the requirement for (precursors of) such functional or bioactive compounds that can be important for health and performance phenotypes. It would be beneficial to not only evaluate the strict-nutritional values of protein sources, but also consider their potential non-strict-nutritional functional properties and effects *in vivo*. However, approaches to evaluate the functional properties of proteins sources beyond their capacity to provide (essential) AA and other nutrients (10) have not been reported.

Measuring amine metabolites in serum or urine is considered a useful approach to assess the host and microbiota metabolism of proteins in the diet. On one hand, a profile of blood amine metabolites would reveal information about the nutritional efficiency of different protein sources and on the other hand, the metabolic and absorptive capacity of the gut, including the influence of the microbiome (11). Systemic amine metabolic profiles are anticipated to be good candidate biomarkers to predict dietary protein-associated phenotypes in relation to health (12–14) but have not been previously studied in relation to different protein sources. Ultimately amine metabolic profiles might reveal biomarkers that could be used to assess health status and nutritional quality of different protein sources.

The aim of this research study was to investigate the effect of mice fed different protein sources, intended for livestock, on the metabolic amine profiles in serum and urine and on host metabolism. Groups of mice were fed semi-purified diets containing different protein sources (300 g/kg) as follows: soybean meal (SBM), casein (CAS), partially delactosed whey powder (DWP), spray-dried plasma protein (SDPP), wheat gluten meal (WGM), and yellow mealworm (YMW) larvae (*Tenebrio molitor*, YMW). The used quantities of protein intake are relevant for animal nutrition. Serum and urine were collected after 14 and 28 days for measurement of a large panel of amine metabolites.

## Materials and Methods

### Diets

Customized semisynthetic diets based on AIN-93G were prepared replacing CAS with one of following alternative protein sources: SBM, DWP, WGM, SDPP, and YMW at an inclusion level of 300 g/kg. Representative samples of dried and ground diets were chemically analyzed for dry matter (NEN-ISO 6496 by 4 h drying at 104°C), nitrogen (N; NEN-ISO 5983-2 by Kjeldahl method and crude protein calculated as N × 6.25), ash (NEN-ISO 5984 after 3 h ashing at 550°C), ether extract (NEN-ISO 6492 by extraction with petroleum ether), and gross energy (NEN-EN-ISO 9831 by bomb calorimetry). The ingredient and chemical composition of the experimental diets is presented in Data Sheet S2, Table S1 in Supplementary Material.

### Animal and Design of Experiment

All procedures were approved by the Wageningen Animal Ethics Committee (Wageningen, The Netherlands; accession number 2012062.c) and carried out according to the guidelines of the European Council Directive 86/609/EEC dated November 1986. A schematic representation and detailed description of the experimental design and sample collection is given in Data Sheet S2, Figure S1 in Supplementary Material. Briefly, upon arrival 72 twenty-one-day-old wild-type male C57BL/6J mice (Harlan Laboratories, Horst, The Netherlands) were stratified according to bodyweight and litter of origin into 6 diet groups in a light and temperature-controlled animal facility of Wageningen University & Research (12:12 h reversed light/dark cycle, 20 ± 2°C). The mice were housed in pairs in a specific pathogen-free environment with *ad libitum* access to diet and water. Prior to the start of the experiment, mice were adapted for 1 week to a standard diet based on AIN-1993 growth (AIN-93G), which included 300 g/kg CAS as the only protein source (as fed basis, CAS). Thereafter, one group continued with the CAS diet, and the other five groups received similar semisynthetic diets containing 300 g/kg (as fed basis) of one of the alternative protein sources [diet containing SBM; diet containing partially DWP; diet containing spray-dried plasma protein (SDPP); diet containing WGM and diet containing YMW] for 28 days. Feed intake and water consumption were measured every day, and body weight of the animals was measured every week. Urine samples were collected just before euthanasia, from each animal by mechanical stimulation of the ventral body section. Six mice from each group were anesthetized with isoflurane and sacrificed at days 14 and 28 to collect blood and urine samples. After euthanasia of the mice, blood samples were collected by orbital puncture, and serum was extracted using 500 µL SST tubes (Becton Dickinson, Franklin Lakes, NJ, USA) within 30 min after collection of the blood. Urine and blood samples were stored at −80°C for further analysis of metabolites.

### Metabolomics Profiling

#### Assay Description

The amine profiling was performed as described previously (15). Briefly, 5 µL of each sample was spiked with an internal standard solution (Table S2 in Supplementary Material), thiol amines were released from proteins and converted to reduced form using tris-(2-carboxyethyl)phosphine. Then proteins were precipitated by the addition of methanol. The supernatant was transferred to an Eppendorf tube (Eppendorf, Germany) and dried in a speedvac (Eppendorf, Germany). The residue was reconstituted in borate buffer (pH 8.5) with AQC reagent (Waters, Etten-Leur, The Netherlands). After reaction, the vials were transferred to an autosampler tray (Waters, Etten-Leur, The Netherlands) and cooled to 10°C prior to injection. For amine metabolite analysis, 1 µL of the reaction mixture was injected into the ultra-performance liquid chromatography mass spectrometry system using an Accq-Tag Ultra column (Waters, Etten-Leur, The Netherlands).

#### Equipment

We employed an ACQUITY UPLC system with autosampler (Waters, Etten-Leur, The Netherlands) was online coupled with a Xevo Tandem Quadrupole (TQ) mass spectrometer (Waters, Etten-Leur, The Netherlands) operated using QuanLynx data acquisition software (version 4.1; Waters, Etten-Leur, The Netherlands). The Xevo TQ was used in the positive-ion electrospray mode, and all analytes were monitored in multiple reaction monitoring (MRM) using nominal mass resolution.

#### Data Processing and Quality Check (QC) of Metabolomics Data

Acquired data were evaluated using TargetLynx software (Waters, Etten-Leur, The Netherlands), by integration of assigned MRM peaks and normalization using proper internal standards. For analysis of AAs, their 13C15N-labeled analogs were used. For other amines, the closest-eluting internal standard was employed (Data Sheet S2, Table S2 in Supplementary Material). Blank samples were used to correct for background, and in-house-developed algorithms were applied using the pooled QC samples to compensate for shifts in the sensitivity of the mass spectrometer over the batch analysis (16). Out of 68 targeted amine metabolites, we could detect 41 amines in serum and 53 amines in urine that comply with the acceptance criteria of QC corrections (16). These metabolites were detected in both serum and urine in each six mice per treatment for days 14 and 28 samples. However, only in CAS, DWP, and SDPP urine samples from day 28, metabolites were detected in five mice per treatment group due to lack of sufficient sample from one animal in each of these groups. The data are represented as relative response ratios (amine target area/area of internal standard; unit free) of these metabolites (after QC) are available in the Data Sheet S1 in Supplementary Material.

### Data Analysis

Univariate statistical analysis was employed to experimental data to examine the effect of dietary treatment. In addition, MetaboAnalyst 3.0 (17), a web-based tool was employed to carry out comprehensive metabolomics data analysis and visualization (http://www.metaboanalyst.ca/).

#### Univariate Statistical Analysis

Results of feed intake, water consumption, and body weight are presented as means ± SEM. Statistical analysis was performed by one-way ANOVA followed by a *post hoc* test (Dunnett test: compared all treatment vs. SBM group as control) using GraphPad prism version 5.03 for Windows Vista (GraphPad Software, San Diego, CA, USA). Statistical significance was defined as *P* value <0.05. SBM diet served as reference to make comparisons with other dietary treatments for the univariate analysis (18, 19). Pearson’s linear correlation was used to calculate the *r* value between the concentrations of apparent ileal digestible (AID) essential amino acids (EAAs) in the experimental diets and the AA concentrations in serum on day 28.

#### Amine Metabolomics Data Analysis

To analyze the amine metabolomics data, MetaboAnalyst 3.0 was employed. Within MetaboAnalyst 3.0, we used two modules, i.e., exploratory (multivariate and clustering) statistical analysis and functional (pathway) analysis. Within the exploratory statistical analysis module, partial least squares discriminant analysis (PLS-DA) method was used for clustering and classification of the treatments based on the amine profile in serum and urine samples from days 14 and 28 of the experimental animals. Unlike principal component analysis, PLS-DA is a robust form of analysis, directed toward factor space that are associated with high variation in the responses but biased toward directions that are accurately predicted (in this case, sample groups, i.e., experimental diets) (20). Here, we have used leave-one-out cross-validation method to measure the predictability performance (*Q*^2^) to validate the PLS-DA model. *Q*^2^ has no standard of comparison or critical value for inferring significance, aside from its theoretical maximum of 1 or an empirically inferred acceptable value of ≥0.4 for a biological model (21). The value of *Q*^2^ closer to 1 is best, whereas above 0.5 is considered good (22). In addition, we have employed permutation tests to overcome the problem of PLS-DA’s propensity to data overfitting that cannot be detected through cross-validation (21). The aim of the permutation-based validation is to measure the performance of the predictor model by determining the probability (*P* value) of observing an equal or better performance by pure chance. For example, if none of the permuted classes is better than the observed 1 in 2,000 permutations, the *P* value is reported as *P* < 0.0005 (less than 1/2,000). Permutation test was carried out with separation distance (between/within) with 2,000 permutations as featured in the MetaboAnalyst 3.0. Briefly, we used the amine metabolic profiles of individual mice from each treatment groups in both serum and urine samples from days 14 and 28 of the experimental period. The SBM diet served as reference for this study and hence, data were normalized by a pooled sample from the SBM group, and log transformation was carried out.

Two outputs, i.e., the score and the loading plots of PLS-DA were used for clustering and classification of the treatments based on the amine profile in day 28 serum samples from the experimental animals. The scores plot provides an intuitive summary of the sample clustering patterns by projecting high-dimensional metabolomics data into two dimensions in a way that explains the maximal covariance (PLS-DA) of the data; while the loading plot shows the underlying compounds responsible for such separation patterns. Two-dimensional (2D) scores plot with 95% confidence region of treatment specific cluster was used to visualized the score plots. In addition, clustered heatmap was used as a tool to find cluster of amine metabolites associated with treatment groups. This provides a visual description of the evolution of the clusters based on the concentration of the metabolites in the treatment groups (23). We choose “do not recognize-samples” to show the natural contrast among treatment groups, and rest other selection criteria or options are left at default for MetaboAnalyst 3.0.

Pathway analysis module of MetaboAnalyst 3.0 was employed to determine the amine metabolism that was affected by the treatments compared to the SBM diet. To perform the pathway analysis, we used the amine metabolic profile of individual mice in serum of day 28 of the experimental period, to prepare the data matrix of individual comparisons of experimental diets with SBM. All the compound names of the metabolites were matched with the human metabolome database. Normalization was performed as described for exploratory statistical analysis. Thereafter, *Mus musculus* pathway library and a reference metabolome based on our technical platform were uploaded (Data Sheet S1 in Supplementary Material, worksheet 6). The analysis includes pathway enrichment analysis, and topological analysis was involved. The impact-value threshold calculated from topology analysis was set at 0.4 and −log (*P*) value calculated from pathway enrichment was set to 4 to identify the most related metabolic pathway.

Variable importance in the projection (VIP) score, another output from above mentioned PLS-DA (based on serum of day 28 of the experimental period) was used to determine the amine metabolites that can discriminate all treatments. The VIP score positively reflects the metabolite’s influence on the classification. Amine metabolomics data analysis were performed on the data as relative response ratios (amine target area/area of internal standard; unit free) of each metabolites retrieved after QC correction, which is available in Data Sheet S1 in Supplementary Material.

## Results

### Composition of the Diets

The experimental diets were formulated to contain adequate levels of EAA, minerals, vitamins, and fatty acids, as recommended for a rodent diets by the American Institute of Nutrition (AIN-93, growth) (24) (Data Sheet S2, Table S1 in Supplementary Material). The concentrations of AID AAs (g/kg) in the diets were calculated using data on the AID of AAs or protein of the protein sources in pigs (Table 1) (25).

**Table 1:** Calculated concentrations of apparent ileal digestible AAs (g/kg) in the experimental diets.^a^

	Diets					
	SBM	CAS	DWP	SDPP	WGM	YMW
ALA	4.9	7.6	2.6	7.6	6.0	NA
ARG	10.0	9.1	1.5	12.5	8.3	8.6
ASP	13.6	18.0	5.7	18.0	7.3	NA
CYS	1.6	0.8	1.2	7.4	5.0	8.6
GLU	22.0	54.2	9.8	54.2	80.3	NA
GLY	4.5	4.4	0.6	4.4	7.3	NA
HIS	3.3	7.9	1.2	7.0	4.9	5.3
ILE	5.7	12.8	3.4	5.9	8.6	9.9
LEU	9.5	24.7	6.0	20.5	16.3	15.8
LYS	8.9	21.3	6.1	20.1	4.8	10.4
MET	4.7	10.6	4.0	4.4	6.7	14.6
PHE	6.6	13.3	2.2	12.0	12.1	6.1
PRO	5.7	27.9	2.9	27.9	29.0	8.0
SER	5.9	13.1	2.5	13.1	10.8	NA
THR	4.6	10.4	3.5	11.3	5.6	NA
TRP	1.7	3.2	0.9	3.2	2.0	7.5
TYR	4.7	14.4	1.5	12.8	7.8	2.4
VAL	5.8	16.5	3.1	13.9	9.2	11.5

*^a^Apparent ileal digestibility of AAs (based on pigs) calculated with digestibility coefficients of AAs in SBM, CAS, DWP, and WGM as listed by CVB (25). Gross AA composition is shown here for YMW-based diet. Here, SBM, soybean meal; CAS, casein; DWP, delactosed whey powder; SDPP, spray-dried plasma protein, WGM, wheat gluten meal; YMW, yellow mealworm; AA, amino acid*.

### Feed Intake and Growth Parameters

All mice appeared to be healthy throughout the experimental period of 4 weeks. However, mean body weight was significantly lower (*P* < 0.05) for the DWP-fed compared to the SBM-fed mice throughout the experimental period (Data Sheet S2, Figure S2 in Supplementary Material). Compared to SBM-fed mice, a significant increase of water consumption was observed in DWP- and WGM-fed mice. Water consumption was significantly lower for CAS-, SDPP-, and YMW-fed mice compared to SBM-fed mice. In YMW-fed mice, we measured a significantly lower feed and water intake compared to the SBM-fed mice (Data Sheet S2, Figure S2 in Supplementary Material), while the development of body weight over the experimental period did not differ compared to the SBM-fed mice.

### Clustering of Metabolites According to Diet

**Figure 1:**
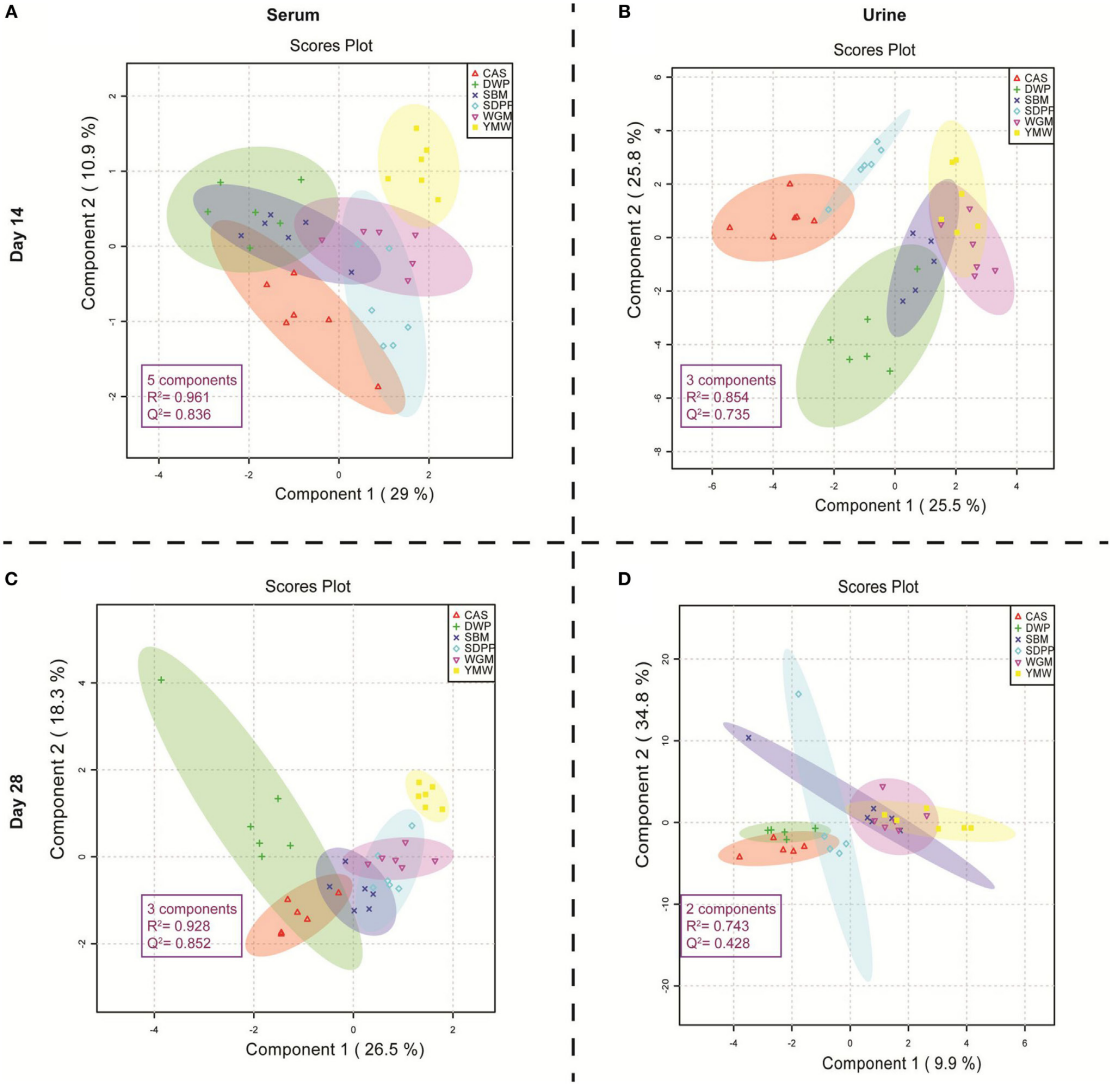
Partial least square discriminant analysis (PLS-DA) score plots of amine profiles in serum and urine on days 14 and 28 of mice receiving diets with different protein sources. **(A)** PLS-DA model built using serum amine metabolites of mice at day 14 of the experimental period; **(B)** PLS-DA model built using urine amine metabolites of mice at day 14 of the experimental period; **(C)** PLS-DA model built using serum amine metabolites of mice at day 28 of the experimental period; **(D)** PLS-DA model built using urine amine metabolites of mice at day 28 of the experimental period. The colored dots represent samples from different treatments. Colored spherical areas display 95% confidence region of respective experimental diets. Each symbol represents data from one mouse. Parameters within the magenta box represent the summary from cross-validation of PLS-DA model. The “components” describe the best number of components the model utilizes to capture the discrimination within treatment group based on the highest *Q*^2^ value in the cross-validation of PLS-DA model. *R*^2^ represents the quality and *Q*^2^ represents the predictability of each PLS-DA model. SBM, soybean meal; CAS, casein; DWP, partially delactosed whey powder; SDPP, spray-dried plasma protein; WGM, wheat gluten meal; YMW, yellow mealworm.

Figure 1 shows the metabolic profile separation of the experimental treatments in 2D PLS-DA, considering score plots of the first two components of the PLS-DA. The PLS-DA score plots, based on the amine metabolites measured in serum on days 14 and 28, had a higher *Q*^2^ value of predictability in relation to the protein source generating the metabolic profile (Figures 1A,C) than the metabolic profiles in urine samples collected on the same days (Figures 1B,D). The predictability performance of the PLS-DA model that is constructed with serum amine metabolites at day 28 to discriminate dietary treatments is high, based on the observed *P* value in the permutation test (Data Sheet S2, Figure S3 in Supplementary Material). Therefore, we subsequently focused on comparing serum amine metabolic profiles from different protein sources on day 28 (Figure 1C). The YMW group metabolite profile was clearly separated from those obtained from the other groups (Figure 1C). The DWP diet also showed a tendency to cluster separately, although an overlap with CAS and SBM was observed using a 95% confidence limit for the shaded plots. The serum amines responsible for the separation of YMW amine metabolic profiles from those to other protein sources, is explained by large amounts of 1-methylhistidine (1-MHis), glycine and 4-hydroxyproline (Data Sheet S2, Figure S4 in Supplementary Material). The serum amine metabolites responsible for the separation of the DWP in the PLS-DA plot 1C were (large amounts) of alpha-aminobutyric acid, S-methylcysteine, threonine, citrulline, and alanine (Data Sheet S2, Figure S5 in Supplementary Material) and (low amounts) of leucine, valine, phenylalanine, glutathione, beta-alanine, and putrescine (Data Sheet S2, Figure S6 in Supplementary Material).

### Correlation between Concentrations of Dietary and Plasma AAs

**Figure 2:**
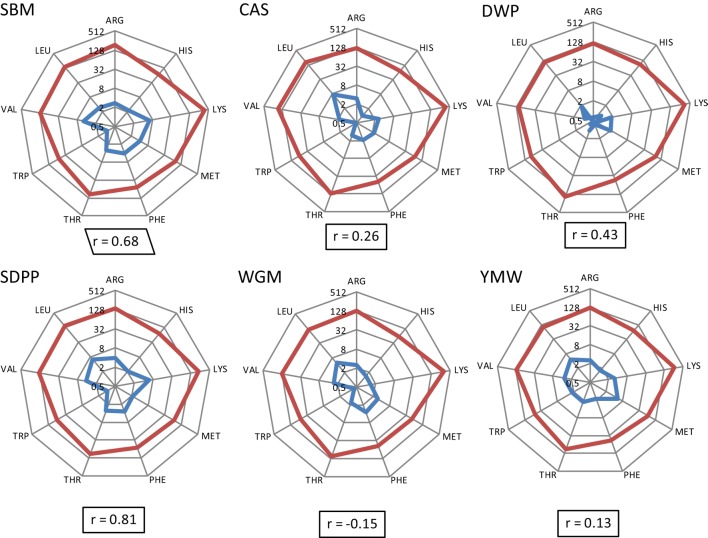
Radial representation of log-transformed calculated concentrations of apparent ileal digestible EAA in the experimental diets and the concentrations of free EAA in serum of mice fed the corresponding experimental diets (day 28). Number on circles refers to essential AA values in logarithmic scale base 2 (log_2_), and the integers in the spider graph are the log-transformed values. Blue line: essential AA composition (g/kg) as determined in the experimental diets. Red line: essential AA composition (μM/mL) as measured in serum on day 28. “*r*” means correlation values between calculated EAA in the experimental diets and the absolute concentrations of EAA in serum of mice. SBM, soybean meal; CAS, casein; DWP, partially delactosed whey powder; SDPP, spray-dried plasma protein; WGM, wheat gluten meal; YMW, yellow mealworm; AA, amino acid; EAA, essential amino acid.

The calculated concentrations of AID EAA in the experimental diets and the measured concentrations of EAA in serum are detailed in Data Sheet S2, Table S3 in Supplementary Material. The correlation coefficients of the apparent dietary EAA and the serum concentrations of EAA are shown per diet in Figure 2. Based on the correlation values (*r*), diets prepared with different protein sources could be ranked as SDPP > SBM > DWP > CAS > YMW > WGM; *r* value ranging from 0.81 to −0.15 (strongest and positive correlation for SDPP, *r* = 0.72; weakest and negative correlation for WGM, *r* = −0.09; Figure 2).

### Diet-Associated Amine Metabolic Sets

**Figure 3:**
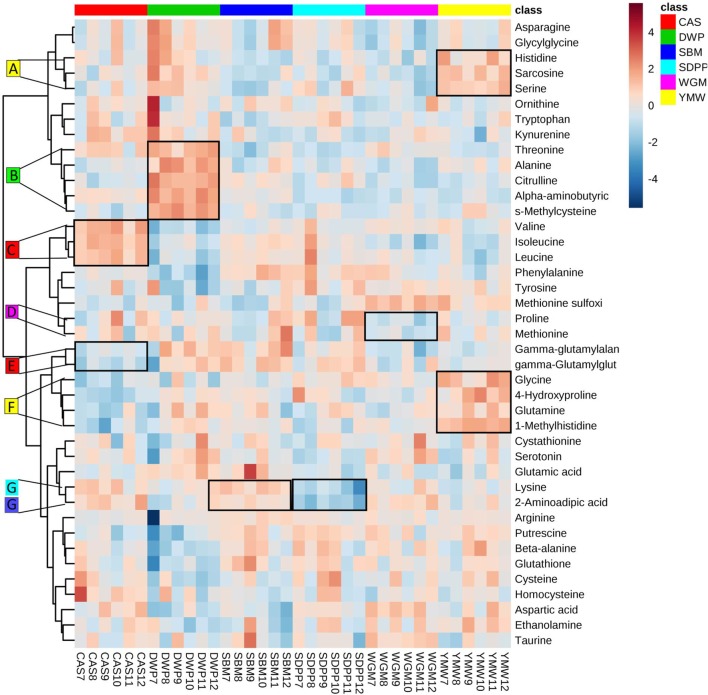
Heatmap of clustering of serum amine metabolites in dependence of dietary treatment. Values are the relative response ratios of each metabolite in individual mice per treatment group. Treatment groups comprise six mice. Values are measured in serum of mice at day 28 of the experimental period. The heatmap graphic distances were measured using Euclidean distances and the clustering algorithm using ward dendrogram. Each colored cell on the map corresponds to a concentration value (normalized to the SBM treatment). The alphabets A–G represent the diet-associated cluster formed by sets of metabolites (framed in black) having low (shades of blue) or high (shades of orange) concentration in the dietary treatments. Here, the colored square corresponds to the respective experimental diets; i.e., red for CAS, green for DWP, navy blue for SBM, sky blue for SDPP, magenta for WGM, and yellow for YMW. SBM, soybean meal; CAS, casein; DWP, partially delactosed whey powder; SDPP, spray-dried plasma protein; WGM, wheat gluten meal; YMW, yellow mealworm. The alphabets A–G indicate amine metabolite clusters referred to in the text.

The clustered heatmap revealed seven (A–G) diet-associated clusters of pathway-related amine metabolites in serum of mice at day 28 of the experimental period (Figure 3). All diets had one or two clusters of amine metabolites that were present in relatively higher or lower concentrations compared to other diets. For example, cluster A comprises histidine, sarcosine, and serine that were found at relatively high concentrations in the YMW-fed mice, and cluster B comprising threonine, alanine, citrulline, alpha-aminobutyric acid, and S-methylcysteine were present at relatively high concentrations in DWP-fed mice.

### Functional Analysis: Metabolic Pathways

**Figure 4:**
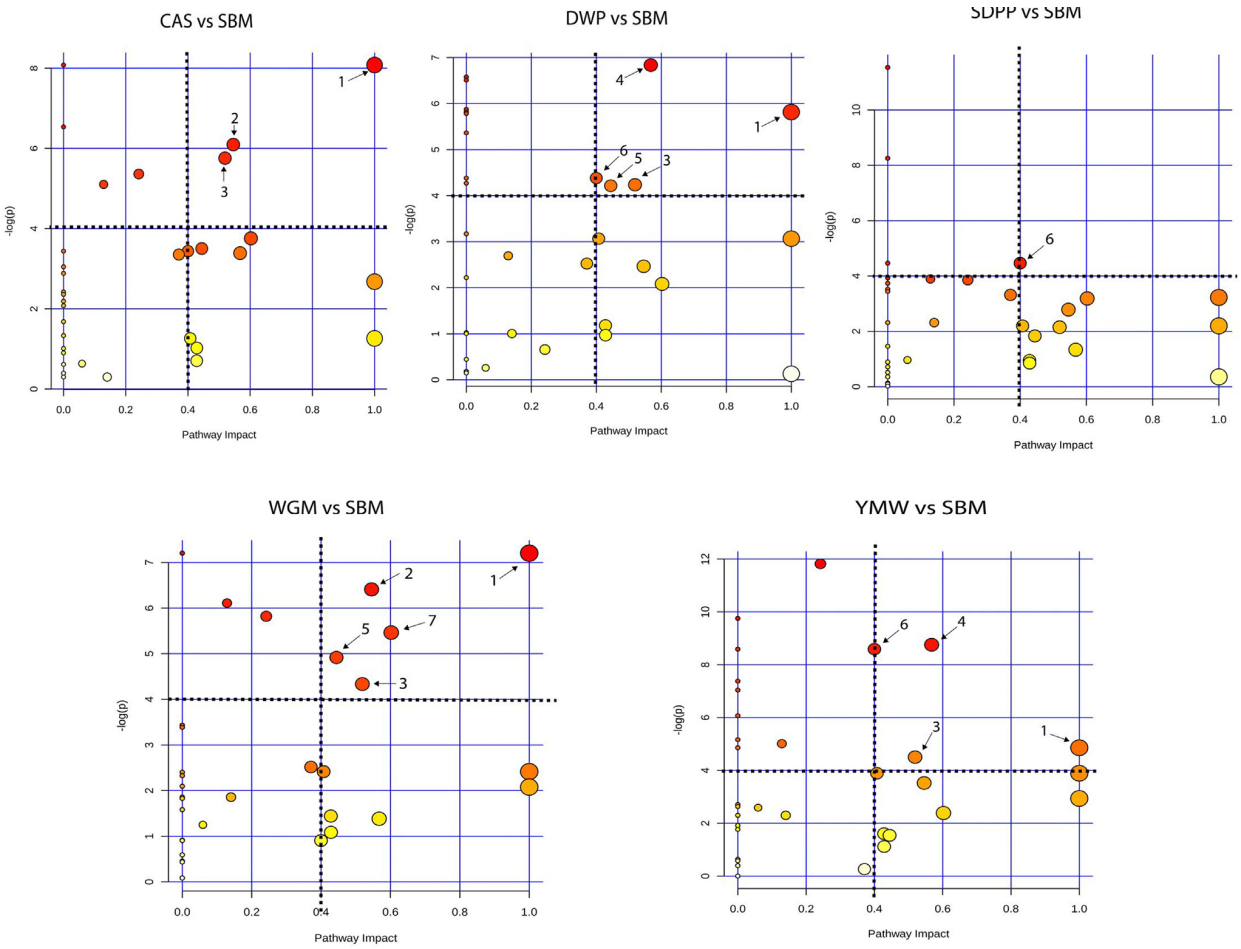
Metabolic pathway analysis based on the plasma amine profiles in mice fed diets with different protein sources. Bubble graph representing the result of metabolic pathway analysis. Scores from enrichment analysis is represented on the “*y*-axis” and from topology analysis on the “*x*-axis.” The size and the color (on gradient scale: white to red) of the nodes are all matched pathways according to *P* values from pathway enrichment analysis and pathway impact values from pathway topology analysis. The dotted black lines denote the thresholds in both axes to identify the most significant matched pathways for all the dietary comparisons. The arrow indicates to the enriched pathways, where, 1—valine, leucine, and isoleucine biosynthesis; 2—arginine and proline metabolism; 3—glutathione metabolism; 4—glycine, serine, and threonine metabolism; 5—beta-alanine metabolism; 6—methane metabolism, and 7—alanine, aspartate, and glutamate metabolism. SBM, soybean meal; CAS, casein; DWP, partially delactosed whey powder; SDPP, spray-dried plasma protein; WGM, wheat gluten meal; YMW, yellow mealworm.

Serum amine metabolites that differed in concentration between SBM and the other protein diets were mapped to Kyoto Encyclopedia of Genes and Genomes (KEGG) metabolic pathways. This revealed seven metabolic pathways that were influenced by dietary protein source (Figure 4). Comparing CAS vs SBM, the enriched KEGG pathways were: valine, leucine, and isoleucine biosynthesis; arginine and proline metabolism and glutathione metabolism. For DWP vs SBM, the enriched KEGG pathways were as follows: valine, leucine, and isoleucine biosynthesis; glutathione metabolism; glycine, serine, and threonine metabolism; beta-alanine metabolism and methane metabolism. For SDPP vs SBM, the only enriched KEGG pathway was methane metabolism. For WGM vs SBM, the enriched KEGG pathways were as follows: valine, leucine, and isoleucine biosynthesis; arginine and proline metabolism; glutathione metabolism; beta-alanine metabolism and alanine, aspartate, and glutamate metabolism. For YMW vs SBM, the enriched KEGG pathways were as follows: valine, leucine, and isoleucine biosynthesis; glutathione metabolism; glycine, serine, and threonine metabolism and methane metabolism (Figure 4). The valine, leucine, and isoleucine biosynthesis pathway was commonly enriched in all diets comparisons except in the SDPP vs SBM comparison. In the pathway, differences were observed in the concentration of the branched chain amino acids (BCAAs, i.e., valine, leucine, and isoleucine) between CAS, WGM, DWP, and YMW (Data Sheet S2, Figures S7–S10 in Supplementary Material).

### Diet-Associated Sets of Amine Metabolites

**Figure 5:**
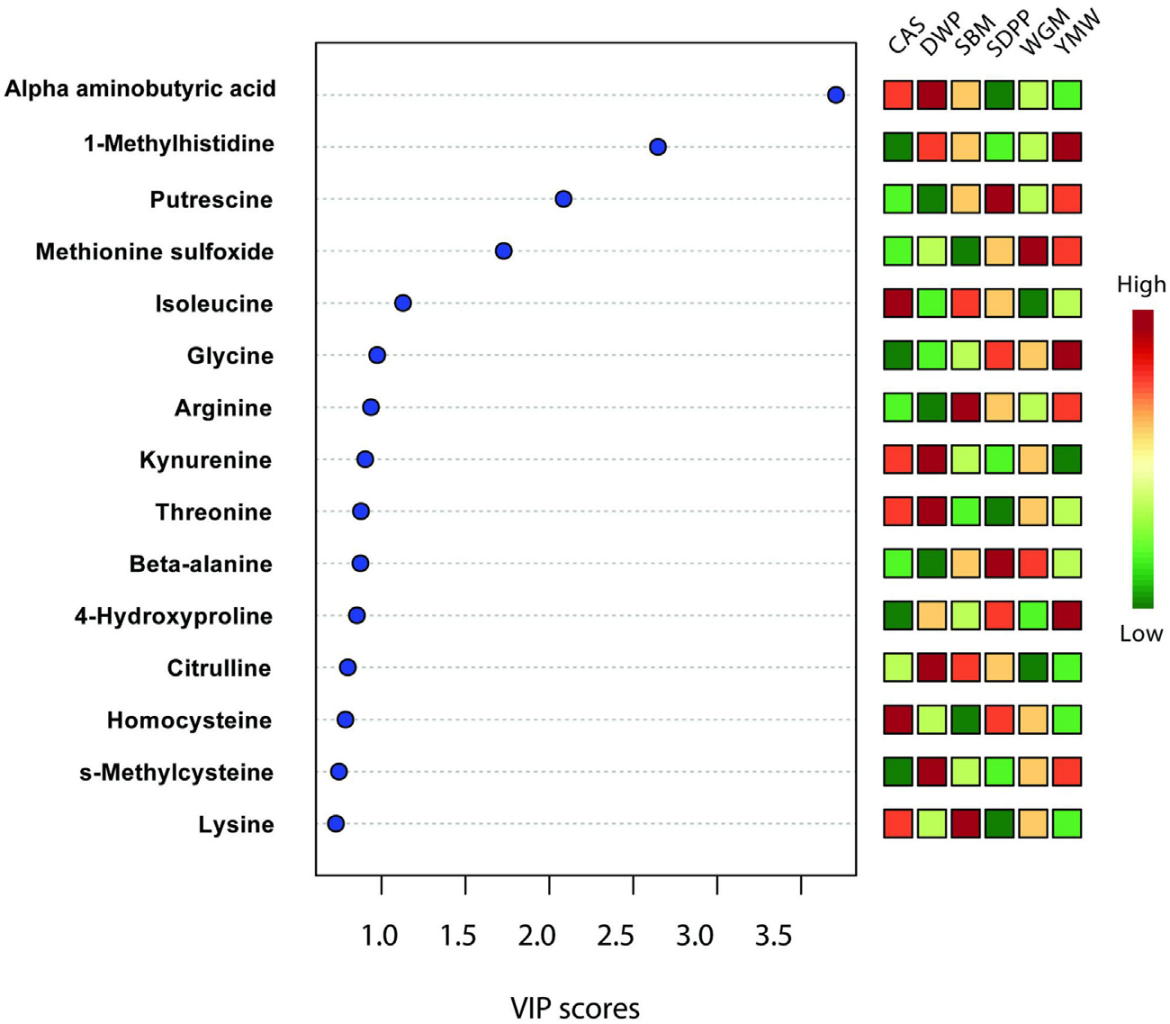
Important features identified by partial least square discriminant analysis model based on amines metabolites in serum at day 28 of experimental period. Metabolites with a high VIP score are predictive for certain diet, e.g., alpha-aminobutyric acid is predictive for DWP and 1-methylhistidine for YMW. The color boxes on the right indicate the relative concentration of the corresponding metabolites in each group under study. CAS, casein; DWP, partially delactosed whey powder; SBM, soybean meal; SDPP, spray-dried plasma protein; WGM, wheat gluten meal; YMW, yellow mealworm; VIP, variable importance in the projection. Values are the mean of six replicates.

Partial least square discriminant analysis plots were used to find the fundamental relations between different diets and amine metabolites in serum or urine (Figure 1). The importance of each variable, i.e., amine metabolite in the projection represented for each diet in the PLS-DA plots is estimated by the VIP score and metabolites having VIP scores greater than 2 can be considered as marker for a specific dietary protein source (26, 27). Three metabolites had VIP scores >2 in the PLS-DA analysis (Figure 5); these were alpha-aminobutyric acid, 1-MHis, and putrescine. A high concentration of alpha-aminobutyric acid and a low concentration of putrescine were observed for DWP-fed mice. Moreover, the dietary treatments can be discriminated based on differences in concentrations of the top seven metabolites. A high concentration of alpha-aminobutyric acid and a low concentration of putrescine were observed in the DWP-fed mice. Similar but reciprocal observations were noticed for alpha-aminobutyric acid and putrescine in the SDPP-fed mice. A high concentration of 1-MHis was observed in YMW-fed mice and a low concentration in the CAS-fed mice.

## Discussion

Here, we show that the inclusion of different protein sources in the diets lead to the appearance of specific amine metabolite profiles in the serum and urine of mice. Differences in these metabolic endophenotypes were most pronounced for the YMW- and DWP-based diet. This might be due to the lower body weight gain for the DWP group and/or the lower feed and water intake in the YMW group and/or provision of AAs in these diets.

**Figure 6:**
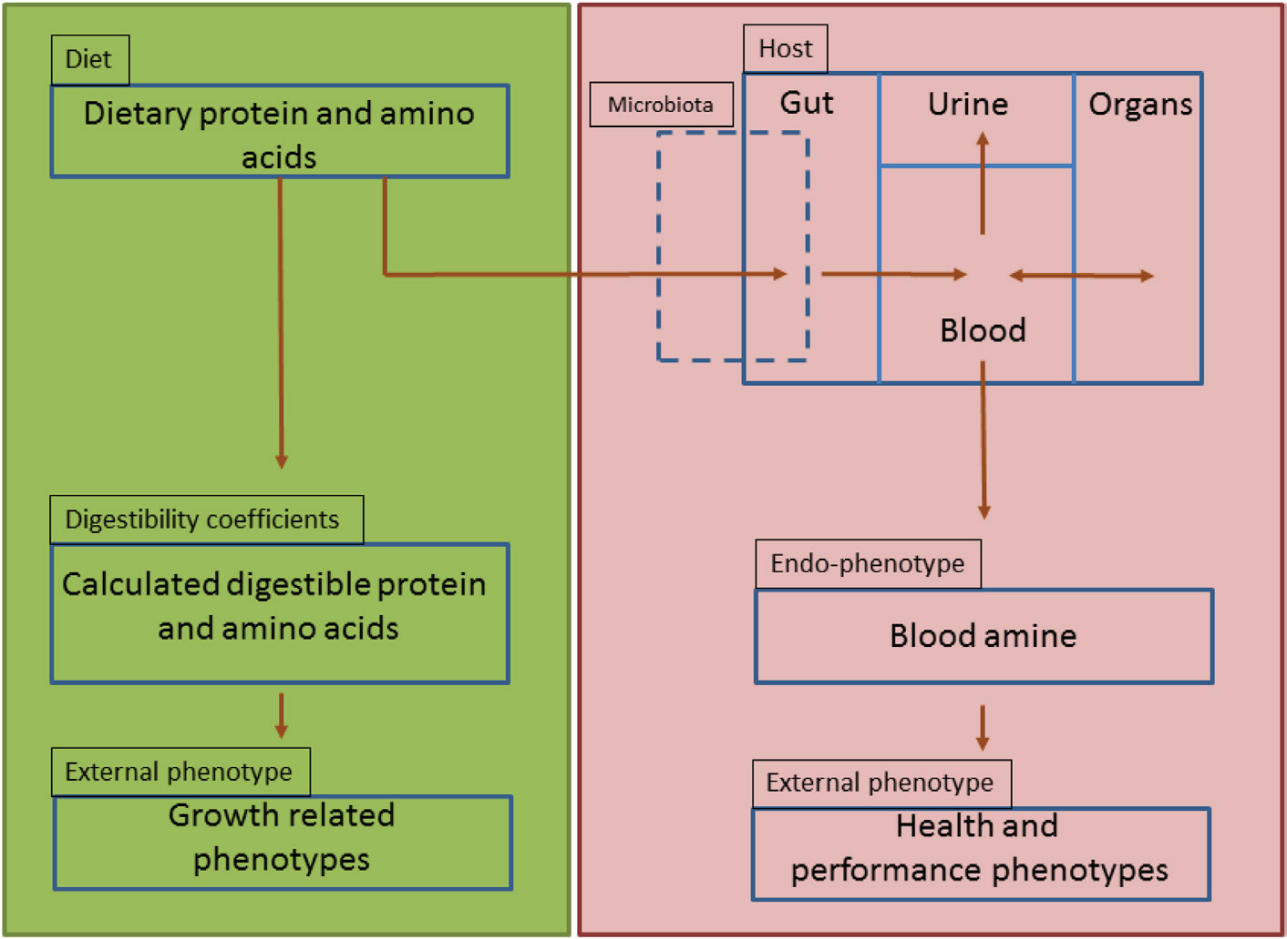
Schematic representation of amine-based endophenotype concept to evaluate dietary protein. Green panel represents the current dietary protein evaluation practice that is based on amino acids and their digestibility coefficients as function of only performance-related phenotypes (strict-nutritional characteristics). Red panel represents the proposed use of amine-based endophenotype in blood for dietary protein source evaluation.

Amine-based profiles or endophenotypes originate from interactions of dietary protein components with the genotype of the animal, its associated microbiome and the production environment (Figure 6). The results described in this paper clearly demonstrate that amine metabolic profiles in blood and urine are affected by the source of dietary protein as it interact with the host and the residing microbiome within the gut environment. These amine profiles are composed of essential and non-essential AAs, amine intermediate metabolites, which are important for cellular protein synthesis but also biosynthesis of neurotransmitters (e.g., serotonin) and immunomodulatory metabolites (e.g., kynurenine). In addition, amines generated by microbial metabolism (e.g., putrescine) and precursors for other nitrogen based biologically active motifs (e.g., glutathione) are also identified using this metabolic platform. Serum amine metabolic profiles were more reliable for discrimination of the dietary protein source than those from urine. This seems logical as amines entering the circulation from the GIT, can be further metabolized or stored in tissue depots of splanchnic organs or undergo further metabolic degradation elsewhere in the body, whereas urine amine metabolites represent excreted amines that are either toxic or metabolically in excess and not required by the host (Figure 6).

The differences in nutrient composition of the diets used in the present study are related to the fixed inclusion (300 g/kg) of different protein sources that contain protein as well as other constituents such as carbohydrates and fats (9). From a nutritional perspective, dietary protein has no nutritional value unless it is hydrolyzed by proteases and peptidases into free AAs, dipeptides, or tripeptides in the lumen of the small intestine (28) and subsequently absorbed and metabolized by the intestinal tissue or transmitted into the bloodstream. In this context, it was interesting to observe that the correlation between dietary and blood essential AAs differed between the used diets. Therefore, we conclude that EAA concentrations in blood are not only related to the quantitative provision of AAs from the diets, based on calculated values for AID AAs, but also dependent on host-, microbiota-, and/or environment-related factors. Apparently, the correlation for the SDPP (*r* = 0.81) and SBM (*r* = 0.68) fit better with the resulting blood profile of the EAA than YMW (*r* = 0.13) and WGM (*r* = −0.15) based diets. This indicates that the EAA profile of SDPP and SBM better mimics the required AA profile by (farm) animals than YMW or WGM.

From the heatmap (Figure 3), we learned that the variation in amine profiles was due to variation in the concentration of metabolically related clusters of metabolites. For example, a set of metabolites formed by valine, leucine, and isoleucine, all showed a higher concentration in the CAS diet compared to other experimental diets. The differential abundance of metabolite clusters in animals fed specific diet suggests a dietary induced regulation of metabolic pathways rather than the regulation of single metabolic steps. Furthermore, we observed an enrichment of the valine, leucine, and isoleucine biosynthesis pathway, based on the amine profile in the serum of mice fed with CAS when compared with SBM. Within the enriched metabolic pathway, the concentration level of valine, leucine, and isoleucine (BCAAs) showed a high concentration in mice fed the CAS diet compared to the SBM diet. In particular, the valine level was significantly (*P* < 0.05) higher in the serum of mice fed the CAS-based diet compared to the SBM-based diet. Our results provide clear evidence that compared to SBM, CAS is an excellent source for BCAA, based upon the combined results of the heatmap and the metabolic pathway analysis. Branched chain AAs have been reported to have health benefit effects because they spare lean body mass during weight loss (29), promote wound healing (30), promote muscle protein anabolism in muscle wasting with aging (31), and have beneficial effects in the setting of renal and liver disease (32). These observations are in agreement with previous findings where a CAS-based diet is capable of influencing AA metabolism mediated *via* BCAA biosynthesis pathway in humans and pigs, compared to soy proteins (33, 34).

As CAS and whey are from milk derived protein source, one may expect that DWP would exhibit a similar metabolic profile to CAS when compared to SBM (35). However, we observed a contrasting result in serum of mice fed with DWP to CAS for AAs profile, in particularly to the BCAA profile (Data Sheet S2, Figure S8 in Supplementary Material). This can be explained by the differences between whey and CAS digestive properties or protein digestion kinetics (36). Studies have shown differences in the peak plasma levels of AAs following ingestion of whey and CAS, i.e., whey protein peaking earlier in time than CAS (37, 38). Our results suggest that, due to the differences in the digestive properties or protein digestion kinetics of DWP (fast) and CAS (slow), these diets differentially alter host protein metabolism.

Based on the difference in the observed amine metabolite profile, we identified two metabolites (namely, alpha-aminobutyric acid for DWP and 1-MHis for YMW) that are highly discriminative for the various amine-based endophenotypes. Alpha-aminobutyric acid is a key intermediate in the synthesis of a tripeptide analog of glutathione (i.e., ophthalmic acid) with antioxidative properties. 1-MHis results from the metabolism of the dipeptide anserine found in meat sources (39, 40). The enzyme carnosinase, present in intestinal mucosal tissues, splits anserine into beta-alanine and 1-MHis. The latter has already been shown as a marker of meat consumption in humans (41). Higher level in YMW-based diet suggests a high dietary supply of anserine from this protein source. To date, there is no evidence to support that 1-MHis itself has any detrimental effects on health (42), but some recent evidences have shown its association with health conditions (43, 44).

The systemic levels of amine metabolites could have predictive value for external phenotypes of livestock animals such as regulation of growth performance, nutritional status, and health characteristics (see Figure 6). On one hand, we observed that both the amine endophenotype as well as the external performance phenotypes varied as function of the used diet. This was most pronounced for the YMW and DWP, as the results of both the amine metabolites as well as the animal body weight gain measurements showed the highest variation among the different diets. On the other hand, we cannot conclude from this study whether amine-based endophenotypes, or (composite) biomarkers derived thereof, can be used to predict external phenotypes associated with specific sources of dietary protein. This will require longitudinal studies in healthy or disease cohorts with a higher number of animals fed diet containing different protein sources. Here, we show that part of (protein) metabolism is influenced by the protein sources included in the diets. Such understanding of metabolism is required to move forward in nutritional science. Future studies may profit from microbiota, gut, and brain metabolites along with immune marker measurements. It would provide improved insight of the effects of dietary protein on the structure of intestinal microbial communities, gut–brain axis signaling, and on immune parameters. In the future, such knowledge may also be useful for predicting the effect of different protein sources on external phenotypes relevant to health and animal performance.

## Ethics Statement

All procedures were approved by the Wageningen Animal Ethics Committee (Wageningen, The Netherlands; accession number 2012062.c) and carried out according to the guidelines of the European Council Directive 86/609/EEC dated November 1986.

## Author Contributions

SK, AJ, DS, JW, and MS contributed to the conception and design of the work. SK drafted the work. SK, DS, LK, and AH contributed to the acquisition, analysis, and interpretation of data. JW, AJ, and MS contributed to the final approval of the version to be publication. All the authors read and approved the final work.

## Conflict of Interest Statement

This study received funding from industrial partners Nutreco and Darling Ingredients Inc. There are no patents, products in development, or marketed products to declare.

## Abbreviations

AID, apparent ileal digestible; BCAA, branched chain amino acid; CAS, casein; DWP, delactosed whey powder; EAA, essential amino acid; GIT, gastro intestinal tract; KEGG, Kyoto Encyclopedia of Genes and Genomes; MHis, methylhistidine; PLS-DA, partial least square discriminant analysis; SBM, soybean meal; SDPP, spray-dried plasma protein; UPLC-MS/MS, ultra-performance liquid chromatography mass spectrometry; VIP, variable importance in the projection; WGM, wheat gluten meal; YMW, yellow mealworm.

## References

[1] van der Spiegel M Noordam MY van der Fels-Klerx HJ Safety of novel protein sources (insects, microalgae, seaweed, duckweed, and rapeseed) and legislative aspects for their application in food and feed production. Compr Rev Food Sci Food Saf (2013) 12(6):662–78. 10.1111/1541-4337.12032 33412718

[2] Boland MJ Rae AN Vereijken JM Meuwissen MPM Fischer ARH van Boekel M The future supply of animal-derived protein for human consumption. Trends Food Sci Technol (2013) 29(1):62–73. 10.1016/j.tifs.2012.07.002

[3] Biesalski H-K Dragsted LO Elmadfa I Grossklaus R Muller M Schrenk D Bioactive compounds: definition and assessment of activity. Nutrition (2009) 25(11–12):1202–5. 10.1016/j.nut.2009.04.023 19695833

[4] Jansman AJM Health and functions of the gastrointestinal tract in pigs: effects of functional ingredients and feed and ingredient processing. J Anim Sci (2016) 94:12–21. 10.2527/jas2015-9886

[5] Saleh S El-Demerdash E. Protective effects of l-arginine against cisplatin-induced renal oxidative stress and toxicity: role of nitric oxide. Basic Clin Pharmacol Toxicol (2005) 97(2):91–7. 10.1111/j.1742-7843.2005.pto_114.x 15998355

[6] Mels CMC Huisman HW Smith W Schutte R Schwedhelm E Atzler D The relationship of nitric oxide synthesis capacity, oxidative stress, and albumin-to-creatinine ratio in black and white men: the SABPA study. Age (Dordr) (2016) 38(1):9 10.1007/s11357-016-9873-6 26767376 PMC5005872

[7] Langen M Kas MJH Staal WG van Engeland H Durston S The neurobiology of repetitive behavior: of mice. Neurosci Biobehav Rev (2011) 35(3):345–55. 10.1016/j.neubiorev.2010.02.005 20156480

[8] Ransford CP. A role for amines in the antidepressant effect of exercise: a review. Med Sci Sports Exerc (1982) 14(1):1–10. 10.1249/00005768-198214010-00001 6280014

[9] Kar SK Jansman AJM Boeren S Kruijt L Smits MA Approximation of the amino acid composition and bio-functional properties of current and novel protein sources for pigs. J Anim Sci (2016) 94:30–9. 10.2527/jas2015-9677

[10] Jahan-Mihan A Luhovyy BL Khoury DE Anderson GH. Dietary proteins as determinants of metabolic and physiologic functions of the gastrointestinal tract. Nutrients (2011) 3(5):574–603. 10.3390/nu3050574 22254112 PMC3257691

[11] Kogut MH Arsenault RJ Editorial: gut health: the new paradigm in food animal production. Front Vet Sci (2016) 3:71 10.3389/fvets.2016.00071 27630994 PMC5005397

[12] Chen TL Xie GX Wang XY Fan J Qiu YP Zheng XJ Serum and urine metabolite profiling reveals potential biomarkers of human hepatocellular carcinoma. Mol Cell Proteomics (2011) 10(7):M110.004945. 10.1074/mcp.M110.004945 21518826 PMC3134066

[13] Picard M McManus MJ Gray JD Nasca C Moffat C Kopinski PK Mitochondrial functions modulate neuroendocrine, metabolic, inflammatory, and transcriptional responses to acute psychological stress. Proc Natl Acad Sci U S A (2015) 112(48):E6614–23. 10.1073/pnas.1515733112 26627253 PMC4672794

[14] Wang Q Wurtz P Auro K Morin-Papunen L Kangas AJ Soininen P Effects of hormonal contraception on systemic metabolism: cross-sectional and longitudinal evidence. Int J Epidemiol (2016) 45(5):1445–57. 10.1093/ije/dyw147 27538888 PMC5100613

[15] Noga MJ Dane A Shi SN Attali A van Aken H Suidgeest E Metabolomics of cerebrospinal fluid reveals changes in the central nervous system metabolism in a rat model of multiple sclerosis. Metabolomics (2012) 8(2):253–63. 10.1007/s11306-011-0306-3 22448154 PMC3291832

[16] van der Kloet FM Bobeldijk I Verheij ER Jellema RH. Analytical error reduction using single point calibration for accurate and precise metabolomic phenotyping. J Proteome Res (2009) 8(11):5132–41. 10.1021/pr900499r 19754161

[17] Xia JG Sinelnikov IV Han B Wishart DS MetaboAnalyst 3.0-making metabolomics more meaningful. Nucleic Acids Res (2015) 43(W1):W251–7. 10.1093/nar/gkv380 25897128 PMC4489235

[18] Heuzé V Tran G Kaushik S Soybean meal. Feedipedia, a Programme by INRA, CIRAD, AFZ and FAO (2016) [updated 27.10.2016].

[19] Gilbert R, editor. World animal feed industry. Protein Sources for the Animal Feed Industry. UN, Rome: FAO, Agriculture and Consumer Protection (2004). p. 1–8.

[20] Barker M Rayens W Partial least squares for discrimination. J Chemom (2003) 17(3):166–73. 10.1002/cem.785

[21] Westerhuis JA Hoefsloot HCJ Smit S Vis DJ Smilde AK van Velzen EJJ Assessment of PLSDA cross validation. Metabolomics (2008) 4(1):81–9. 10.1007/s11306-007-0099-6

[22] Fujimura Y Kurihara K Ida M Kosaka R Miura D Wariishi H Metabolomics-driven nutraceutical evaluation of diverse green tea cultivars. PLoS One (2011) 6(8):e23426. 10.1371/journal.pone.0023426 21853132 PMC3154454

[23] Beckonert O Bollard ME Ebbels TMD Keun HC Antti H Holmes E NMR-based metabonomic toxicity classification: hierarchical cluster analysis and k-nearest-neighbour approaches. Anal Chim Acta (2003) 490(1–2):3–15. 10.1016/s0003-2670(03)00060-6

[24] Reeves PG Rossow KL Lindlauf J Development and testing of the AIN-93 purified diets for rodents – results on growth, kidney calcification and bone mineralization in rats and mice. J Nutr (1993) 123(11):1923–31. 8229309 10.1093/jn/123.11.1923

[25] CVB. Chemical Composition and Nutritional Values of Feedstuffs and Feeding Standards. The Hague, The Netherlands: Product Board Animal Feed (2007).

[26] Palermo G Piraino P Zucht H-D. Performance of PLS regression coefficients in selecting variables for each response of a multivariate PLS for omics-type data. Adv Appl Bioinforma Chem (2009) 2:57–70. 21918616 10.2147/aabc.s3619 PMC3169946

[27] Perez-Enciso M Tenenhaus M. Prediction of clinical outcome with microarray data: a partial least squares discriminant analysis (PLS-DA) approach. Hum Genet (2003) 112(5–6):581–92. 10.1007/s00439-003-0921-9 12607117

[28] Wu GY Dietary protein intake and human health. Food Funct (2016) 7(3):1251–65. 10.1039/c5fo01530h 26797090

[29] Layman DK. The role of leucine in weight loss diets and glucose homeostasis. J Nutr (2003) 133(1):261S–7S. 12514305 10.1093/jn/133.1.261S

[30] Zhang XJ Chinkes DL Wolfe RR. Leucine supplementation has an anabolic effect on proteins in rabbit skin wound and muscle. J Nutr (2004) 134(12):3313–8. 15570031 10.1093/jn/134.12.3313

[31] Rieu I Sornet C Bayle G Prugnaud J Pouyet C Balage M Leucine-supplemented meal feeding for ten days beneficially affects postprandial muscle protein synthesis in old rats. J Nutr (2003) 133(4):1198–205. 12672943 10.1093/jn/133.4.1198

[32] Marchesini G Bianchi G Merli M Amodio P Panella C Loguercio C Nutritional supplementation with branched-chain amino acids in advanced cirrhosis: a double-blind, randomized trial. Gastroenterology (2003) 124(7):1792–801. 10.1016/s0016-5085(03)00323-8 12806613

[33] Luiking YC Deutz NEP Jakel M Soeters PB. Casein and soy protein meals differentially affect whole-body and splanchnic protein metabolism in healthy humans. J Nutr (2005) 135(5):1080–7. 15867285 10.1093/jn/135.5.1080

[34] Deutz NEP Bruins MJ Soeters PB. Infusion of soy and casein protein meals affects interorgan amino acid metabolism and urea kinetics differently in pigs. J Nutr (1998) 128(12):2435–45. 9868192 10.1093/jn/128.12.2435

[35] Otani H Hata I Inhibition of proliferative responses of mouse spleen lymphocytes and rabbit Peyers patch cells by bovine-milk caseins and their digests. J Dairy Res (1995) 62(2):339–48. 10.1017/S0022029900031034 7601978

[36] Boirie Y Dangin M Gachon P Vasson MP Maubois JL Beaufrere B. Slow and fast dietary proteins differently modulate postprandial protein accretion. Proc Natl Acad Sci U S A (1997) 94(26):14930–5. 10.1073/pnas.94.26.14930 9405716 PMC25140

[37] Dangin M Boirie Y Garcia-Rodenas C Gachon P Fauquant J Callier P The digestion rate of protein is an independent regulating factor of postprandial protein retention. Am J Physiol Endocrinol Metab (2001) 280(2):E340–8. 11158939 10.1152/ajpendo.2001.280.2.E340

[38] Bos C Metges CC Gaudichon C Petzke KJ Pueyo ME Morens C Postprandial kinetics of dietary amino acids are the main determinant of their metabolism after soy or milk protein ingestion in humans. J Nutr (2003) 133(5):1308–15. 12730415 10.1093/jn/133.5.1308

[39] Block WD Hubbard RW Steele BF Excretion of histidine and histidine derivatives by human subjects ingesting protein from different sources. J Nutr (1965) 85:419–25. 14273743 10.1093/jn/85.4.419

[40] Sjolin J Hjort G Friman G Hambraeus L. Urinary excretion of 1-methylhistidine: a qualitative indicator of exogenous 3-methylhistidine and intake of meats from various sources. Metabolism (1987) 36(12):1175–84. 10.1016/0026-0495(87)90245-9 3683186

[41] Myint T Fraser GE Lindsted KD Knutsen SF Hubbard RW Bennett HW. Urinary 1-methylhistidine is a marker of meat consumption in Black and in White California Seventh-day Adventists. Am J Epidemiol (2000) 152(8):752–5. 10.1093/aje/152.8.752 11052553

[42] Owusu-Apenten R Bioactive Peptides: Applications for Improving Nutrition and Health. Florida: CRC Press (2010).

[43] Cross AJ Major JM Rothman N Sinha R. Urinary 1-methylhistidine and 3-methylhistidine, meat intake, and colorectal adenoma risk. Eur J Cancer Prev (2014) 23(5):385–90. 10.1097/CEJ.0000000000000027 24681531 PMC4121566

[44] Rafikova O Meadows ML Kinchen JM Mohney RP Maltepe E Desai AA Metabolic changes precede the development of pulmonary hypertension in the monocrotaline exposed rat lung. PLoS One (2016) 11(3):e0150480. 10.1371/journal.pone.0150480 26937637 PMC4777490

