# Supplementary material for: Amine Metabolism Is Influenced by Dietary Protein Source
Source: Front Nutr. 2017 Sep 1;4:41. doi: 10.3389/fnut.2017.00041 (PMC5585152; doi:10.3389/fnut.2017.00041)
Supplement: Supplementary file 2 [file Data_Sheet_2.DOCX]

***Supplementary Material***

**Amine Metabolism is Influenced by Dietary Protein Source**

**Soumya K. Kar, Alfons J.M. Jansman, Dirkjan Schokker, Leo Kruijt, Amy C. Harms, Jerry M. Wells and Mari A. Smits**

*** Correspondence:** Corresponding Author: soumya.kar@wur.nl

**2. Supplementary Figures and Tables**

**2.1 Supplementary Tables**

**Supplementary Table 1. Ingredient and calculated or analysed nutrient composition of the experimental diets for mice, as fed basis**

|  | Diets^1^ | | | | | |
| --- | --- | --- | --- | --- | --- | --- |
| Item | SBM | CAS | DWP | SDPP | WGM | YMW |
| **Ingredient, *g/kg*** |  |  |  |  |  |  |
| Maize | 297.5 | 297.5 | 297.5 | 297.5 | 297.5 | 297.5 |
| Dextrose | 132 | 132 | 132 | 132 | 132 | 132 |
| Sugar | 100 | 100 | 100 | 100 | 100 | 100 |
| Arbocell | 50 | 50 | 50 | 50 | 50 | 50 |
| Soybean Oil | 70 | 70 | 70 | 70 | 70 | 70 |
| AIN-93G MX | 35 | 35 | 35 | 35 | 35 | 35 |
| AIN-93-VX | 10 | 10 | 10 | 10 | 10 | 10 |
| Choline chloride | 2.5 | 2.5 | 2.5 | 2.5 | 2.5 | 2.5 |
| DL-Methionine | 3 | 3 | 3 | 3 | 3 | 3 |
| Soybean meal | 300 | 0 | 0 | 0 | 0 | 0 |
| Casein | 0.0 | 300 | 0 | 0 | 0 | 0 |
| Delactosed whey powder | 0.0 | 0 | 300 | 0 | 0 | 0 |
| Spray dried plasma protein | 0.0 | 0 | 0 | 300 | 0 | 0 |
| Wheat gluten meal | 0.0 | 0 | 0 | 0 | 300 | 0 |
| Yellow meal worm | 0.0 | 0 | 0 | 0 | 0 | 300 |
| **Composition, *g/kg*** |  |  |  |  |  |  |
| Dry matter | 914 | 957 | 930 | 924 | 917 | 929 |
| Crude protein | 153 | 268 | 80 | 238 | 252 | 148 |
| Ash | 43 | 29 | 77 | 28 | 47 | 35 |
| Crude fat | 76 | 65 | 74 | 87 | 70 | 160 |
| Starch | 251 | 249 | 249 | 249 | 268 | 261 |
| Sugar | 295 | 263 | 403 | 263 | 271 | 263 |
| Non starch polysaccharides | 71 | 8 | 17 | 15 | 2 | 18 |
| Gross energy, *KJ/g* | 17 | 19 | 16 | 18 | 18 | 20 |
| Ca | 5.9 | 5.5 | 10.0 | 5.2 | 5.2 | 6.1 |
| P | 3.7 | 3.3 | 6.2 | 1.9 | 2.3 | 4.0 |
| K | 10.4 | 4.0 | 16.5 | 4.5 | 4.0 | 3.6 |
| Na | 1.1 | 1.2 | 5.9 | 8.2 | 1.3 | 1.0 |
| Cl | 1.7 | 2.1 | 10.4 | 12.7 | 1.9 | 1.6 |
| Linoleic acid | 38 | 36 | 36 | 36 | 36 | 36 |
| Electrolyte balance, *Meq/kg* | 266 | 94 | 388 | 115 | 106 | 92 |

^1^Diets: SBM is soybean meal, CAS is casein, DWP partially delactosed whey powder, SDPP is spray dried porcine plasma, SBM is soybean meal, WGM is wheat gluten meal and YMW is yellow meal worm.

**Supplementary Table 2. Internal standards used in the amine profiling platform.**

| **Internal Standards** |
| --- |
| 2-(4-hydroxy-3-methoxyphenyl) ethyl-1, 1,2,2-d4-amine |
| Ala_C13N15 |
| Arg_C13N15 |
| Asn_C13N15 |
| Asp_C13N15 |
| Beta-alanine-2,2,3,3,-d4 |
| Gln_C13N15 |
| Glu_C13 N15 |
| Gly_C13N15 |
| Histamine-a,a,P,P-d4 2HCl |
| L-2-aminobutyric acid-d6 acid |
| L-3-(4-hydroxy-3-methoxy-d3-phenyl)-alanine |
| Leu_C13N15 |
| L-lle C13N15 |
| L-Methionine |
| L-NT-methyl-d3-L-histidine |
| L-ornithine-3,3,4, 4,5,5,-d6 |
| Lys_C13N15 |
| Phe_C13N15 |
| Pro_C13N15 |
| Ser_C13N15 |
| Thr_C13N 15 |
| Trp_C13N15 |
| Tyr_C13N15 |
| Val_C13N15 |

**Supplementary Table 3.** **Calculated concentrations of apparent ileal digestible essential amino acids (EAA) in the experimental diets and the measured concentrations of EAA in serum samples of d 28.**

| Diets | **SBM** | | **CAS** | | **DWP** | | **SDPP** | | **WGM** | | **YMW** | |
| --- | --- | --- | --- | --- | --- | --- | --- | --- | --- | --- | --- | --- |
| Amino Acids | Diet (g/kg) | Serum (uM/ml) | Diet (g/kg) | Serum (uM/ml) | Diet (g/kg) | Serum (uM/ml) | Diet (g/kg) | Serum (uM/ml) | Diet (g/kg) | Serum (uM/ml) | Diet (g/kg) | Serum (uM/ml) |
| ARG | 9.1 | 189.2 | 10.0 | 120 | 1.5 | 114.4 | 12.5 | 138.4 | 8.3 | 129.2 | 7.6 | 8.6 |
| HIS | 7.9 | 73.2 | 3.3 | 73.6 | 1.2 | 89.1 | 7.0 | 74.4 | 4.9 | 61.9 | 4.7 | 5.3 |
| LYS | 21.3 | 408.7 | 8.9 | 396.1 | 6.1 | 341.3 | 20.1 | 238 | 4.8 | 315.1 | 8.1 | 10.4 |
| MET^1^ | 13.6 | 95.9 | 7.7 | 72.2 | 7.0 | 78.3 | 7.4 | 71.3 | 9.7 | 52.5 | 2.6 | 17.6 |
| PHE | 13.3 | 58.2 | 6.6 | 50 | 2.2 | 43.7 | 12.0 | 57.9 | 12.1 | 48.8 | 7.1 | 6.1 |
| THR | 10.4 | 95 | 4.6 | 125.9 | 3.5 | 158.1 | 11.3 | 92.6 | 5.6 | 106.2 | 6.6 | 7.5 |
| TRP | 3.2 | 56.4 | 1.7 | 61.8 | 0.9 | 78.8 | 3.2 | 65 | 2.0 | 54.1 | 2.1 | 7.5 |
| VAL | 16.5 | 126.8 | 5.8 | 172.2 | 3.1 | 104.1 | 13.9 | 134.2 | 9.2 | 125.1 | 10.5 | 11.5 |
| LEU | 9.5 | 142.1 | 24.7 | 163.6 | 6.0 | 109.3 | 20.5 | 146.5 | 16.3 | 115.1 | 15.9 | 15.8 |
| **Corel** | 0.68 | | 0.26 | | 0.43 | | 0.81 | | -0.15 | | 0.13 | |

^1^ Adjusted for 3 g of methionine used to prepare the experimental diets.

**2.2 Supplementary Figures**

**
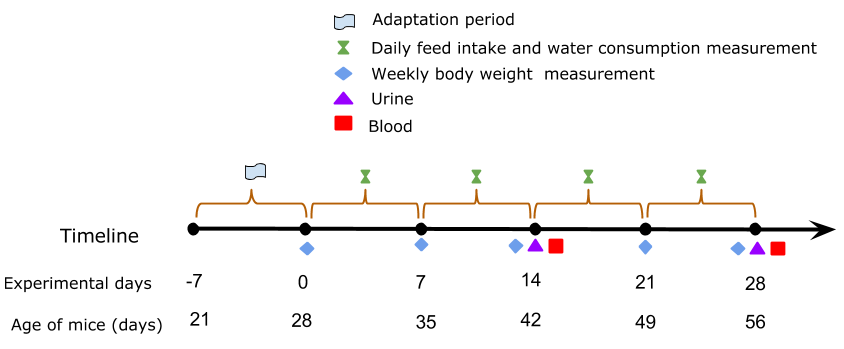
**


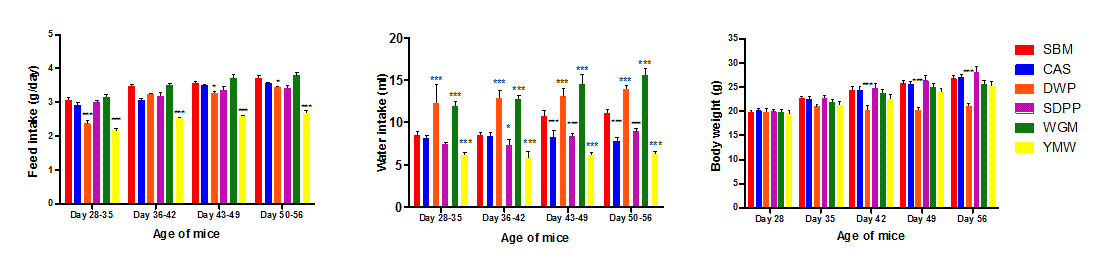
**Supplementary Figure 1.** **Design of the experiment.** The solid black dot in the timeline represents the corresponding experimental days/age of the mice (days). Blood and urine samples were used for amine metabolome analysis.

**Supplementary Figure 2.** **Exophenotype or zoo-technical parameters (feed intake, water intake bodyweight) measurements of mice fed with different experimental diets.** Bars and whiskers represent mean values ± SEM (n = 6), respectively for feed intake (left) and body weight (right) recorded throughout the experimental period. *P < 0.05, ***P < 0.001 compared with SBM-fed mice fed. Here, SBM, soybean meal; CAS, casein preparation intended for animal use; DWP, partially delactosed whey powder; SDPP, spray dried porcine plasma; WGM, wheat gluten meal and YMW, yellow meal worm.

**
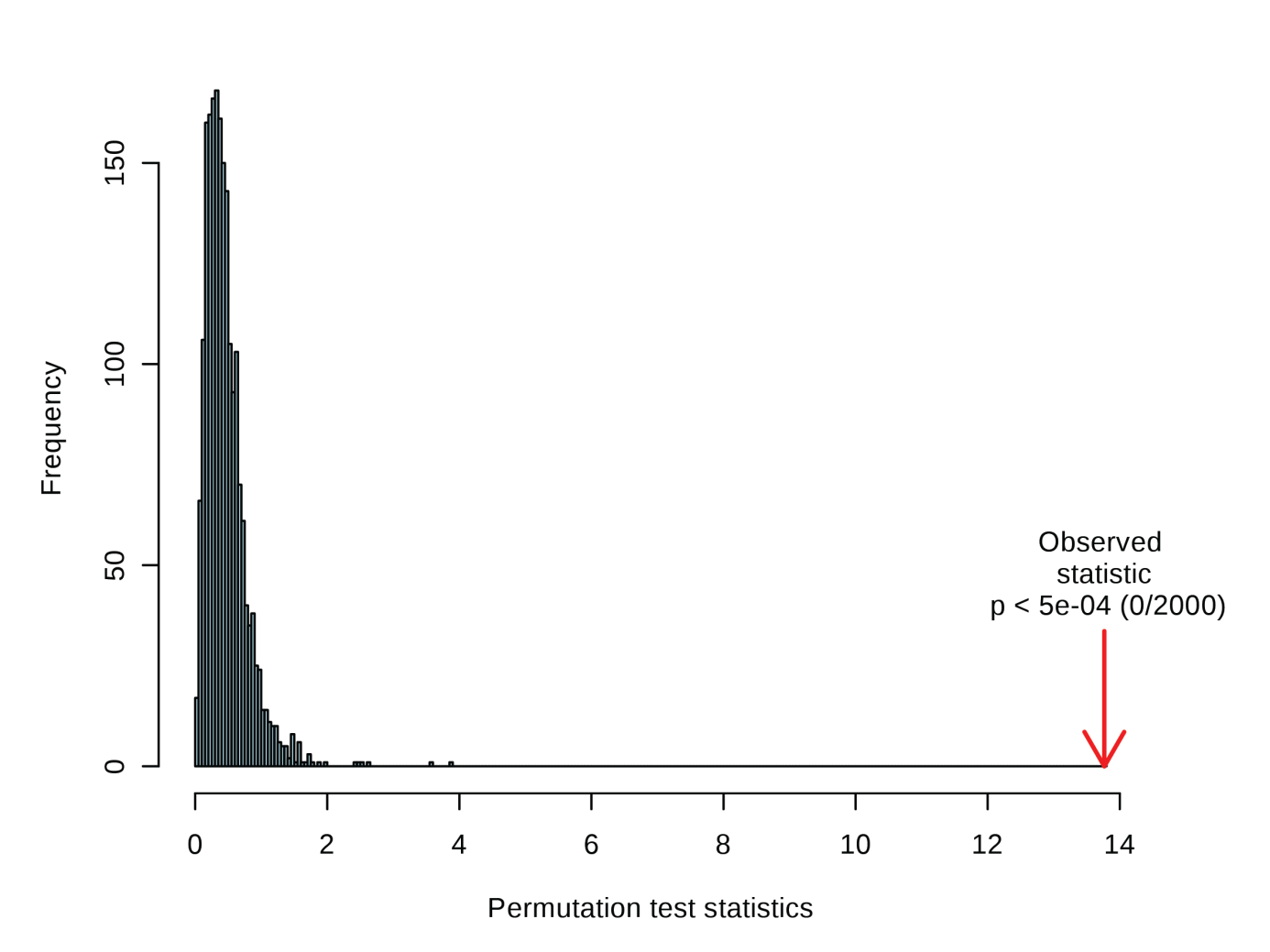
Supplementary Figure 3: The result of permutation test summarised by a histogram for PLS-DA model built from serum amine of mice at d 28 of the experimental period.** Red arrow indicates the empirical p value that is calculated by determining the number of times the permutated data yielded a better result than the one using the original labels.

**
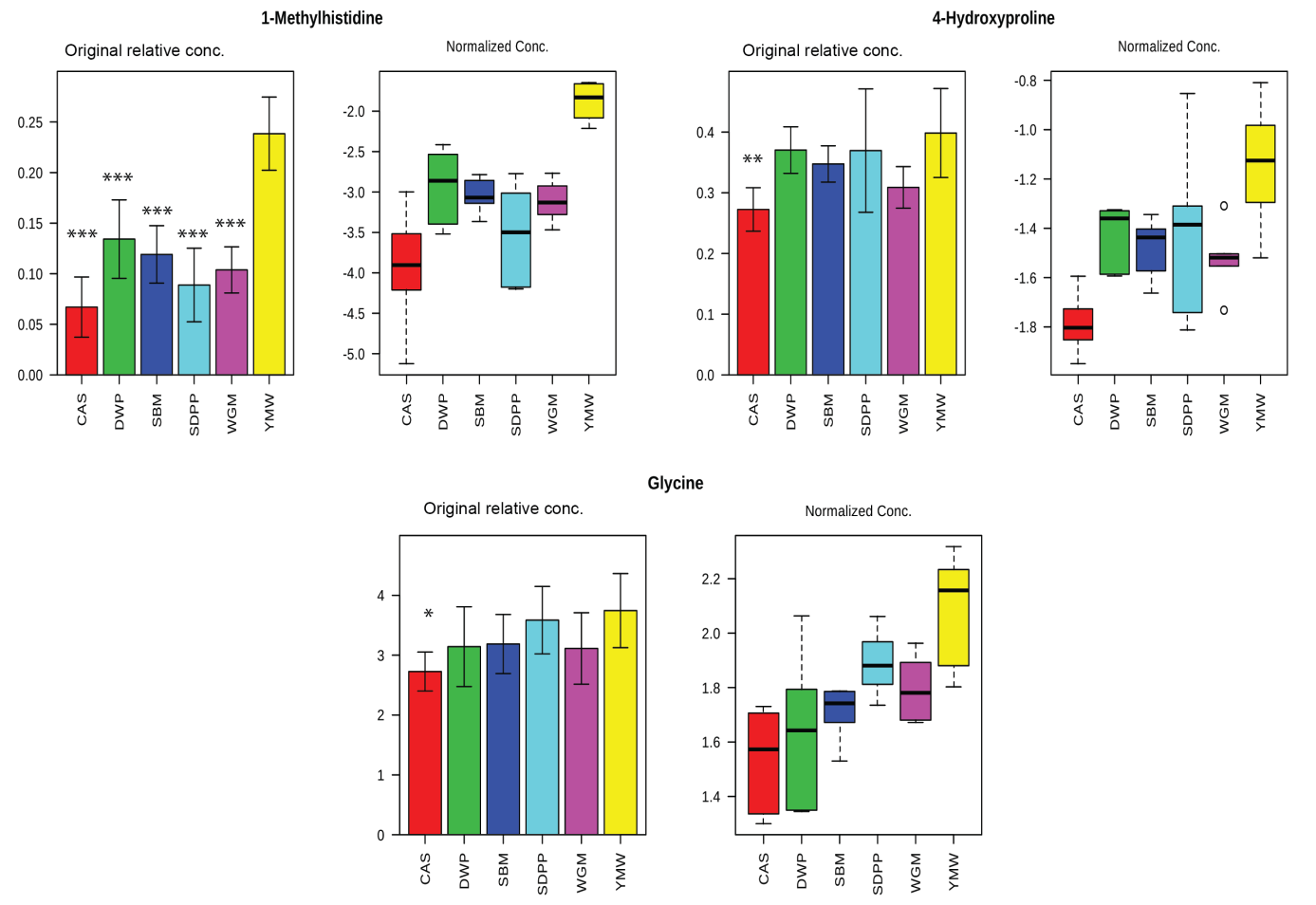
Supplementary Figure 4: Underlying amines which are present relatively in high level in YMW compared to other (at least in one) treatments.** According to loading plot, these featured amine are responsible for separation of YMW group in the PLS-DA model built with d 28 serum amine data. Bars are mean values of the original relative concentration and box are mean values of the normalized concentration in the treatment groups; whiskers are ± Standard Error Mean (n = 6). Statistical analysis was performed in the original concentration (i.e. data as relative response ratios) by one-way ANOVA followed by post hoc test (Dunnett test: compared all treatment vs. YMW group) using GraphPad prism version 5.03 for Windows Vista (GraphPad Software, San Diego, California, USA). Statistical significance was defined as p < 0.05. *P < 0.05, **P<0.01 and ***P < 0.001 compared with YMW group. SBM, soybean meal; CAS, casein preparation intended for animal use; DWP, partially delactosed whey powder; SDPP, spray dried porcine plasma; WGM, wheat gluten meal and YMW, yellow meal worm.

**
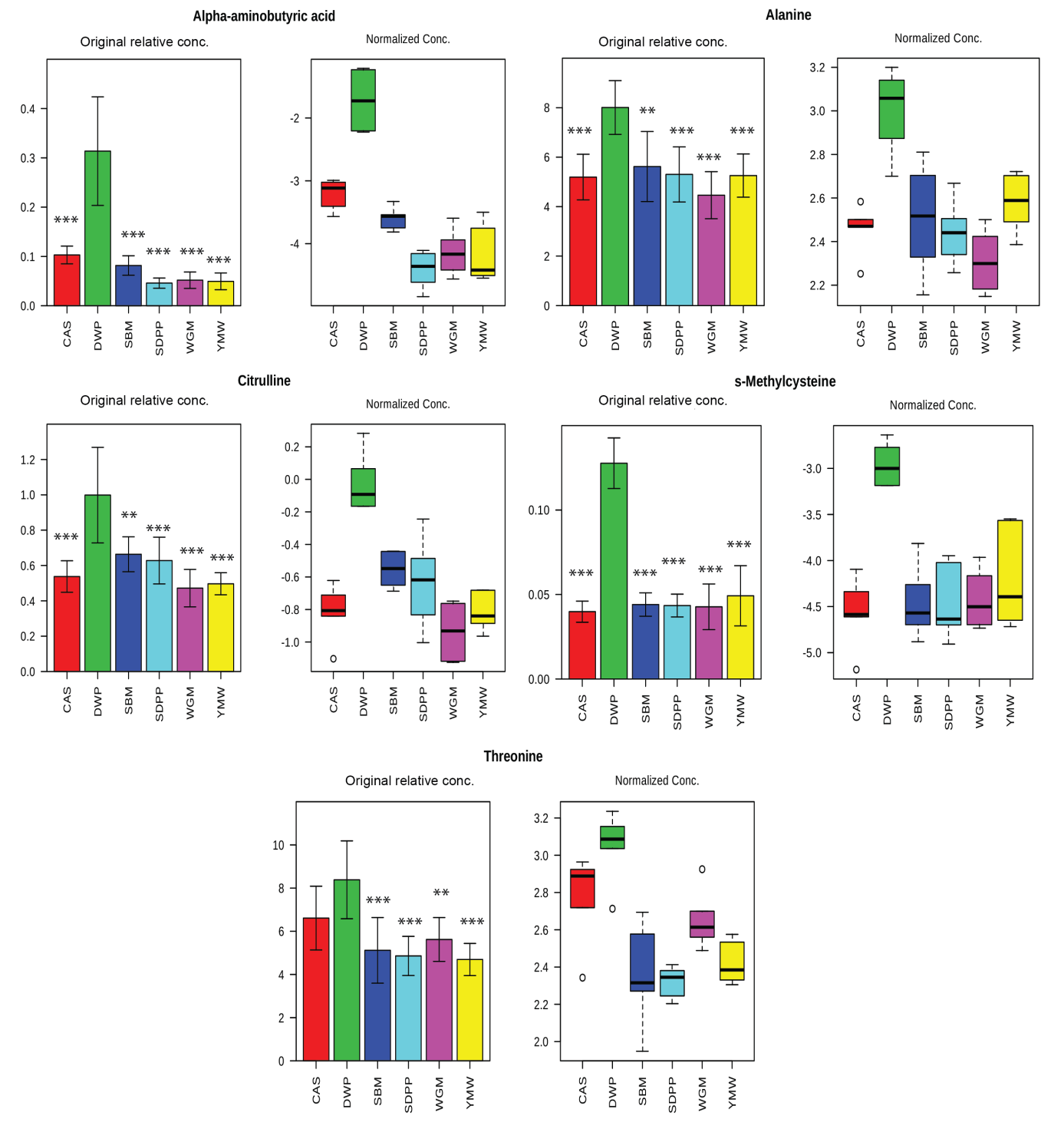
Supplementary Figure 5: Underlying amines which are present relatively in high level in DWP compared to other (at least in one) treatments.** According to loading plot, these featured amine are responsible for separation of DWP group in the PLS-DA model built with d 28 serum amine data. Bars are mean values of the original relative concentration and box are mean values of the normalized concentration in the treatment groups; whiskers are ± Standard Error Mean (n = 6). Statistical analysis (see supplementary figure 4

**
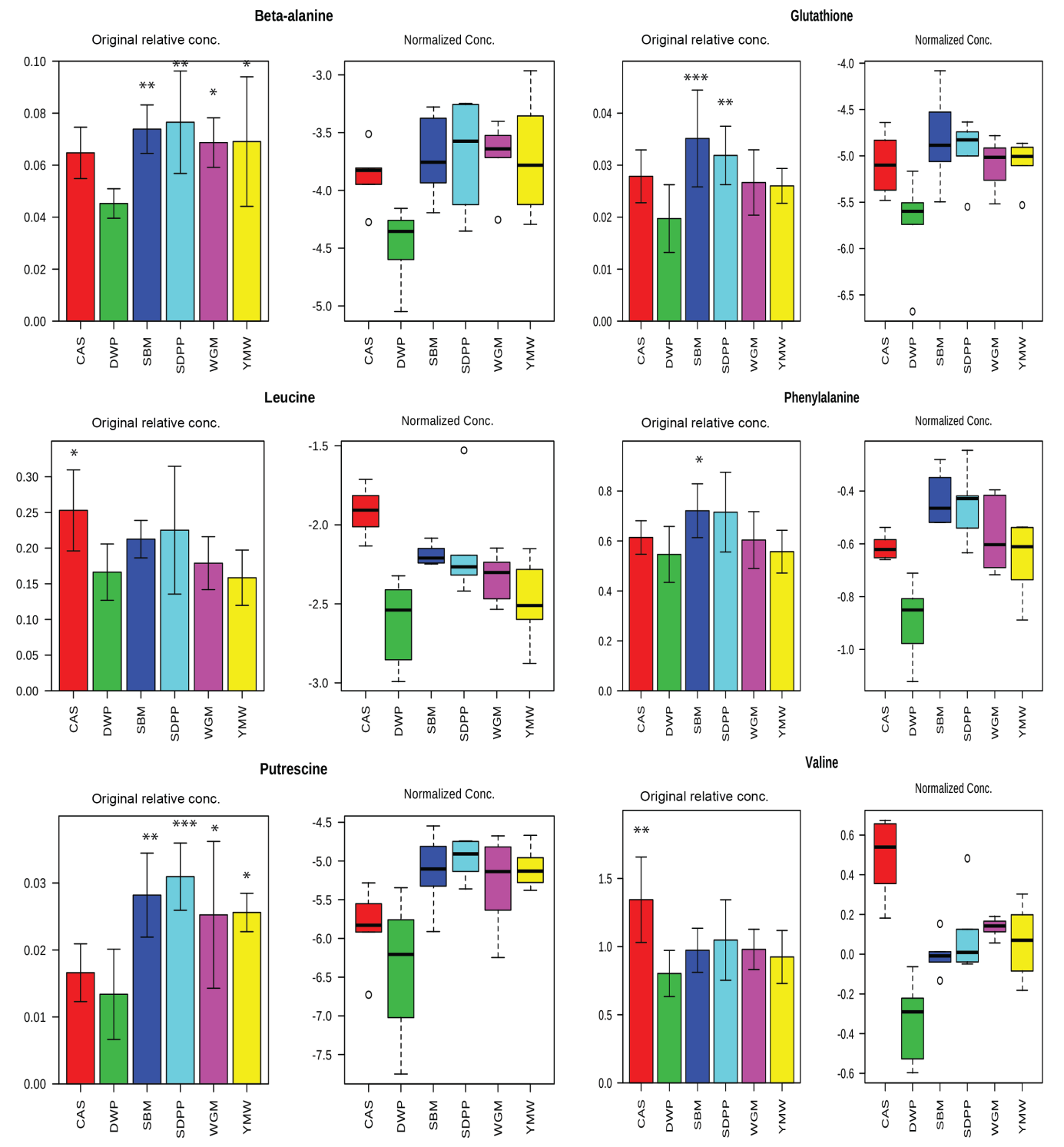
Supplementary Figure 6: Underlying amines which are present relatively in low level in DWP compared to other (atleast in one) treatments.** According to loading plot, these featured amine are responsible for separation of DWP group in the PLS-DA model built with d 28 serum amine data. Bars are mean values of the original relative concentration and box are mean values of the normalized concentration in the treatment groups; whiskers are ± Standard Error Mean (n = 6). Statistical analysis (see supplementary figure 4).

**
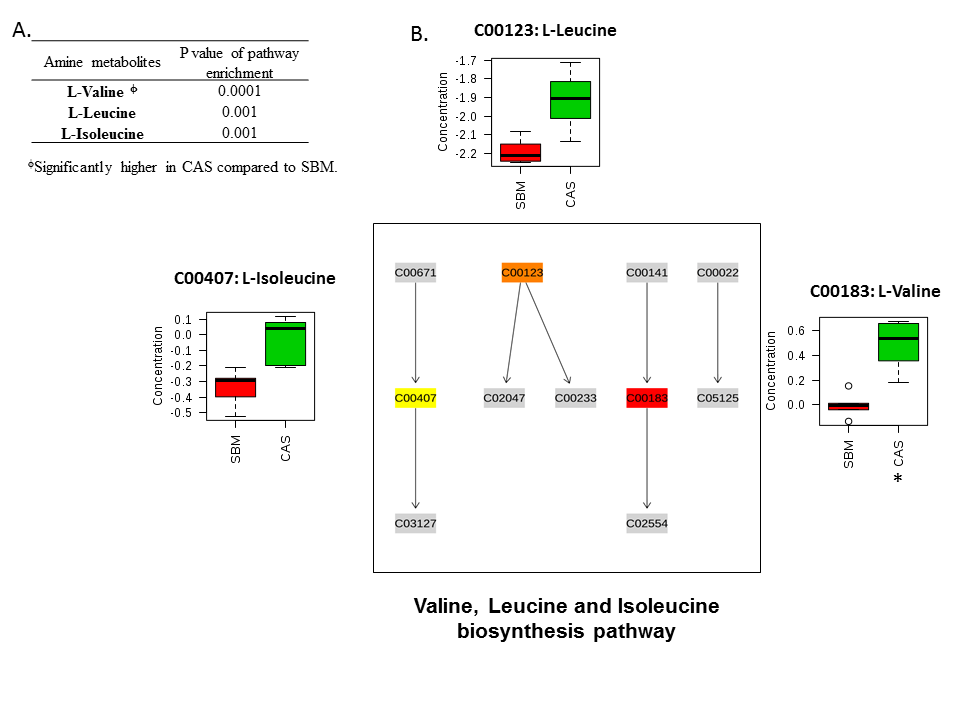
Supplementary Figure 7: Valine, leucine and isoleucine biosynthesis pathway as represented in the Kyoto Encyclopaedia of Genes and Genomes (KEGG) enriched in metabolic pathway analysis based on the amine profile in d 28 serum of mice fed CAS and SBM diet.** A. In bold, amine metabolites that are significantly (P < 0.05) enriched in the pathway enrichment analysis. Valine is significantly (P < 0.05) higher in serum of CAS fed mice than SBM-fed mice. B. The valine, leucine and isoleucine biosynthesis pathway along with it significant enriched amine metabolites. “Alpha-numeric” representations are the KEGG identifiers. The colour (on gradient scale: cream to red, where towards red denotes lower P value) of the rectangular-nodes in the KEGG pathway are all matched amines according to P values from pathway enrichment analysis. The grey rectangular nodes are the un-matched amines in the KEGG pathway. Box plots are mean value of the normalized concentration in the treatment groups; whiskers are ± Standard Error Mean (n = 6 mice per dietary group).

**Supplementary Figure 8: Valine, leucine and isoleucine biosynthesis pathway as represented in the Kyoto Encyclopaedia of Genes and Genomes (KEGG) enriched in metabolic pathway analysis based on the amine profile in d 28 serum of mice fed DWP and SBM diet.** A. In bold, amine metabolites that are significantly (P < 0.05) enriched in the pathway enrichment analysis. None of the enriched metabolites significantly (P < 0.05) differ in DWP from SBM. B. The valine, leucine and isoleucine biosynthesis pathway along with it significant enriched amine metabolites. “Alpha-numeric” representations are the KEGG identifiers. The colour (on gradient scale: cream to red, where towards red denotes lower P value) of the rectangular-nodes in the KEGG pathway are all matched amines according to P values from pathway enrichment analysis. The grey rectangular nodes are the un-matched amines in the KEGG pathway. Box plots are mean value of the normalized concentration in the treatment groups; whiskers are ± Standard Error Mean (n = 6 mice per dietary group).
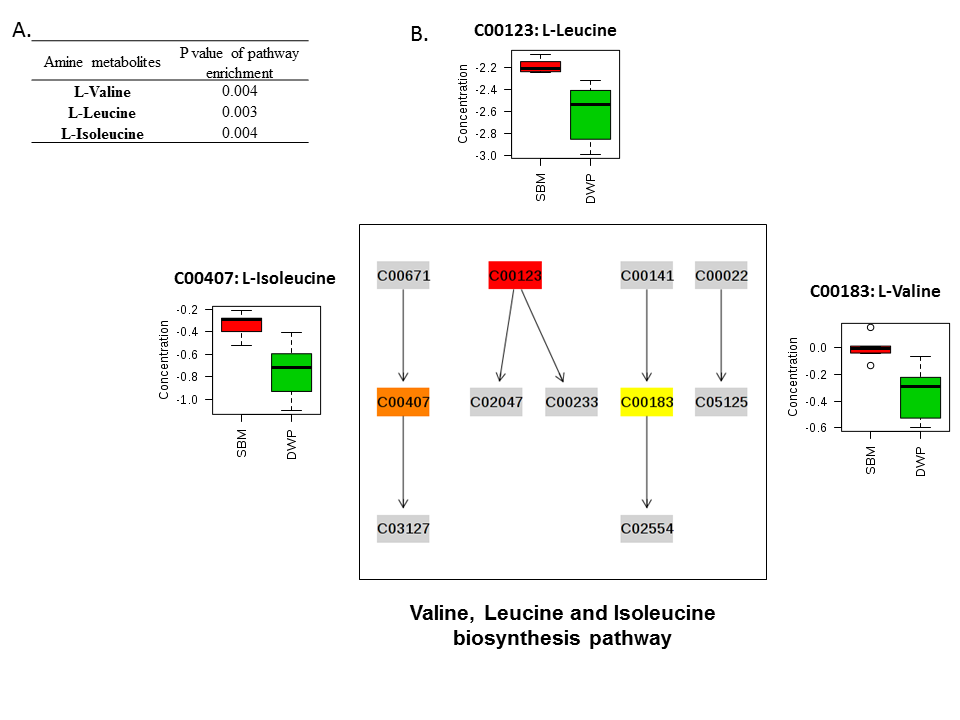


**
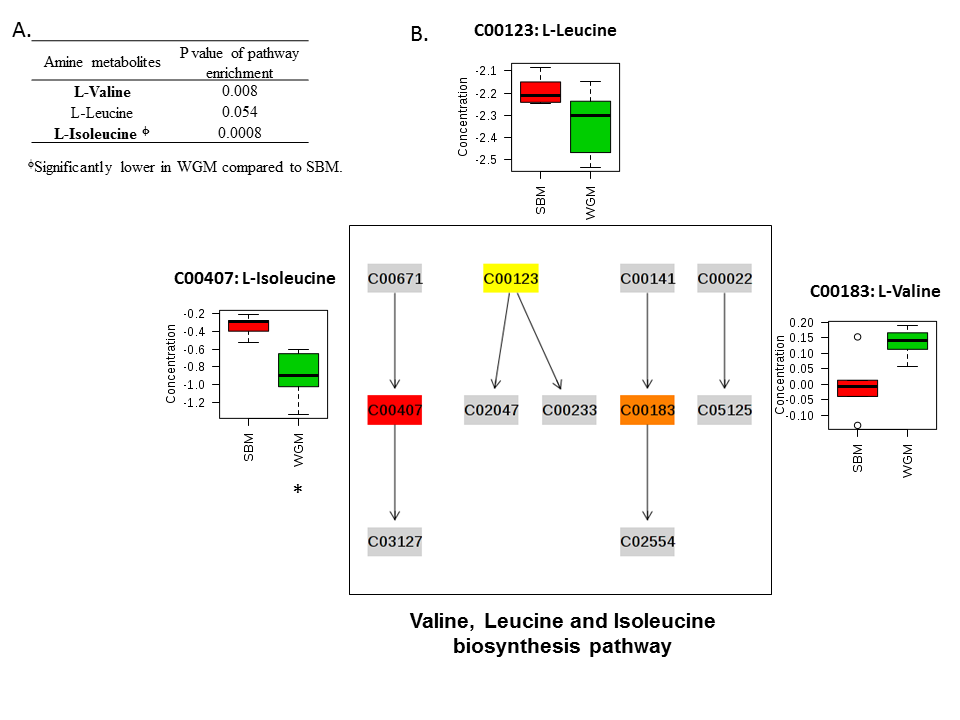
Supplementary Figure 9: Valine, leucine and isoleucine biosynthesis pathway as represented in the Kyoto Encyclopaedia of Genes and Genomes (KEGG) enriched in metabolic pathway analysis based on the amine profile in d 28 serum of mice fed WGM and SBM diet.** A. In bold, amine metabolites that are significantly (P < 0.05) enriched in the pathway enrichment analysis. Isoleucine is significantly (P < 0.05) lower in serum of WGM fed mice than SBM-fed mice. B. The valine, leucine and isoleucine biosynthesis pathway along with it significant enriched amine metabolites. “Alpha-numeric” representations are the KEGG identifiers. The colour (on gradient scale: cream to red, where towards red denotes lower P value) of the rectangular-nodes in the KEGG pathway are all matched amines according to P values from pathway enrichment analysis. The grey rectangular nodes are the un-matched amines in the KEGG pathway. Box plots are mean value of the normalized concentration in the treatment groups; whiskers are ± Standard Error Mean (n = 6 mice per dietary group).

**
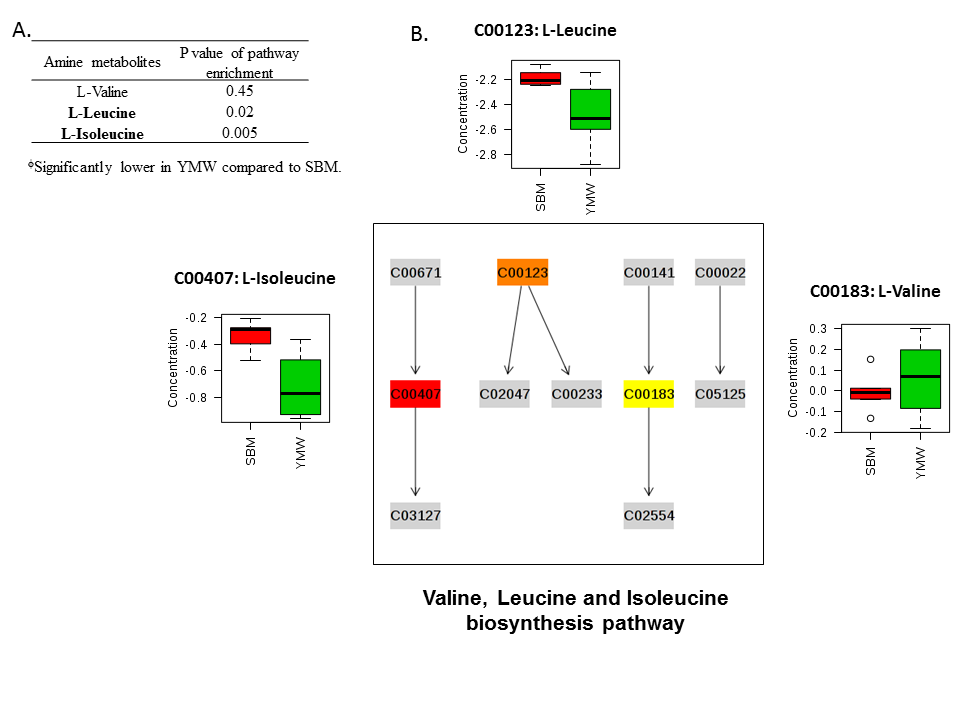
Supplementary Figure 10: Valine, leucine and isoleucine biosynthesis pathway as represented in the Kyoto Encyclopaedia of Genes and Genomes (KEGG) enriched in metabolic pathway analysis based on the amine profile in d 28 serum of mice fed YMW and SBM diet.** A. In bold, amine metabolites that are significantly (P < 0.05) enriched in the pathway enrichment analysis. None of the enriched metabolites significantly (P < 0.05) differ in YMW from SBM. B. The valine, leucine and isoleucine biosynthesis pathway along with it significant enriched amine metabolites. “Alpha-numeric” representations are the KEGG identifiers. The colour (on gradient scale: cream to red, where towards red denotes lower P value) of the rectangular-nodes in the KEGG pathway are all matched amines according to P values from pathway enrichment analysis. The grey rectangular nodes are the un-matched amines in the KEGG pathway. Box plots are mean value of the normalized concentration in the treatment groups; whiskers are ± Standard Error Mean (n = 6 mice per dietary group).
